# Has_circ_0000069 expression in breast cancer and its influences on prognosis and cellular activities

**DOI:** 10.32604/or.2022.028168

**Published:** 2023-03-01

**Authors:** GANG WANG, MINGPING QIAN, WEI JIAN, JUHANG CHU, YIXIANG HUANG

**Affiliations:** Department of General Surgery, Shanghai Tenth People’s Hospital, Tongji University School of Medicine, Shanghai, 200072, China

**Keywords:** Biomarker, Breast cancer, Invasion, Migration, Proliferation

## Abstract

Circular RNA (circRNA), as a newly discovered non-coding RNA with important regulatory potential, is closely related to the occurrence and progression of various tumors. This study aimed to investigate has_circ_0000069 expression in breast cancer and its influence on cellular activities. Using real-time quantitative polymerase chain reaction, has_circ_0000069 levels were measured in 137 pairs of tissue specimens, as well as cancer cell lines. The cellular activities of cell lines were determined by cell counting kit-8 (CCK-8) and Transwell assays. The potential targeting miRNAs were predicted and verified using an online database and dual-luciferase reporter assay. Has_circ_0000069 was highly expressed in breast cancer tissues and cells. The expression of has_circ_0000069 was associated with the five-year overall survival of patients. After silencing has_circ_0000069 in breast cancer cells, its expression reduced, and the ability of cell proliferation, migration, and invasion decreased. MiR-432 was verified as a targeting miRNA of has_circ_0000069. Has_circ_0000069 expression increased in breast cancer and was negatively related to patient’s prognosis. Has_circ_0000069 may facilitate breast cancer tumor progression by sponging miR-432. These findings revealed that has_circ_0000069 may be a biomarker for predicting prognosis and a therapeutic target for treating patients with breast cancer.

## Introduction

Breast cancer, the most common cancer in women worldwide, is a malignant disease that occurs in the epithelial tissue of the breast [1]. The cancer statistics in 2022 showed that the incidence of breast cancer continued to increase from 2014 through 2018 [2]. In China, with the rapid development of the society and economy, breast cancer ranks first and threatens the quality of life of patients [3]. At present, with the continuous development of adjuvant therapies such as molecular targeted therapy and endocrine therapy, the 5-year survival rate has improved [4]. However, tumor recurrence and drug resistance are still major challenges that threaten patients’ overall survival in the treatment of breast cancer [5]. Since the mechanism of breast cancer is very complex, the biomolecular characteristics of breast cancer are diverse, resulting in different treatment effects for patients, and an inability to achieve the ideal treatment state [6]. Therefore, exploring the molecular markers and mechanism of breast cancer development can improve the understanding of tumor biology and this is of great significance for more effective treatment of breast cancer.

Circular RNA (CircRNA) belongs to a special class of non-coding RNAs, which has no 5′ and 3′ end polyadenylate tail and it is a closed circRNA molecule formed by a covalent bond [7]. Increasing studies have proven that circRNA acts a crucial regulatory role in the occurrence and progression of diseases, including malignant tumors [8–10]. CircRNAs are good molecular marker of malignant tumors with high stability, preservation, and specificity. CircRNAs are involved in the pathological and physiological processes of cell growth, differentiation, metastasis, development, and apoptosis of various tumors [11,12]. There are multiple complementary binding sites of miRNAs on circRNAs, which can interact with miRNAs [13]. Several circRNAs are involved in breast cancer progression, such as circ_0025202 [14], has_circ_001783 [15], and circFOXK2 [16]. Has_circ_0000069 is aberrantly expressed in several cancers, such as cervical cancer [17], pancreatic cancer [18], and colorectal cancer [19]. A recent RNA sequencing profile study identified many circRNA-miRNA-mRNA networks, including has_circ_0000069 [20]. The role of has_circ_0000069 in breast cancer remains needs to be explored and its clinical significance needs to verify.

Here, the expression of has_circ_0000069 in tumor tissues and cells was detected and its clinical significance was verified. Further studies investigated the oncogenic regulatory role of has_circ_0000069 in breast cancer cellular behaviors via acting as a ceRNA for miR-432. These findings may provide a putative biomarker for the prognosis and therapy of breast cancer.

## Materials and Methods

### Clinical specimens

A total of 137 pairs of tumor tissue specimens and adjacent normal tissue specimens from breast cancer patients diagnosed by pathology after surgical resection in the Shanghai Tenth People’s Hospital, Tongji University School of Medicine from July 2016 to May 2019 were collected. No patients received preoperative adjuvant therapy. The specimens were stored in liquid nitrogen. All patients signed informed consent before surgery. All specimens were obtained with the approval of the Ethics Committee of the Shanghai Tenth People’s Hospital, Tongji University School of Medicine. The clinicopathological data of patients were obtained and recorded.

### Cell lines and cell culture

Breast cancer cell lines BT549, MCF-7, SK-BR-3, T47D, MDA-MB-231, and normal mammary epithelial cell MCF-10A were obtained from the Type Culture Collection of the Chinese Academy of Sciences (Shanghai, China). Breast cancer cells were cultured in a DMEM medium with 10% FBS (Gibco, Grand Island, NY, USA) and penicillin-streptomycin antibody (Biyuntian, Shanghai, China). MCF-10A cells were cultured with DMEM/F12 (1:1) medium, 5% equine serum (Gibco), 10 μg/mL insulin (Wanbang Biochemical, Jiangsu, China), epidermal growth factor (20 ng/ml; Sigma, Shanghai, China), and 0.5 μg/mL hydrocortisone. The culture condition was an incubator containing 5% CO_2_ at 37°C.

### Cell transfection

When the confluence of cells reached 70%, si-NC(5′-GGACUCUCGGAUUGUAAGAUU-3′), si-circRNA-1(5′-CTACTTCAGGCACAGGTCT-3′), si-circRNA-2 (5′-GCACAGGTCTTCCCAAAAG-3′), or si-circRNA-3 (5′-CTTCAGGCACAGGTCTTC-3′) (Ribobio, Guangzhou, China) was transfected into MCF-7 or MDA-MB-231 cells using lipofectamine 2000. The cells were harvested after 24 h for subsequent experiments.

### RNA isolation and real-time quantitative polymerasechain reaction (RT-qPCR)

Total RNA was isolated from cancer tissues, adjacent tissues, and cell lines by the TRIzol (Invitrogen, Carlsbad, CA, USA) method. A microplate reader was used to measure the concentration and purity of total RNA. Reverse transcription was performed using the PrimeScript^TM^ RT kit (Takara, Tokyo, Japan). RT-PCR was performed using SYBR Premix Ex Taq^TM^ II (Takara, Tokyo, Japan) to detect expression levels of has_circ_0000069 in tissues and cells. Using GAPDH as an internal reference and cDNA as a template, the samples were denatured at 95°C for 3 min, followed by 35 cycles of denaturing at 95°C for 5 s, 60°C for 30 s, and extension at 72°C for 30 s. The 2^−ΔΔCt^ method was used to detect the relative has_circ_0000069 levels. The primers for RT-qPCR were as follows: has_circ_0000069, F: 5′-CTACTTCAGGCACAGGTCTTC-3′; R: 5′-CTGACTCACTGGATGAGGACT3′; GAPDH F: 5′-AAGGTGAAGGTCGGAGTCA-3′; R: 5′-GGAAGATGGTGATGGGATTT-3′.

### Cell proliferation experiments

After 24 h of transfection, breast cancer cells (MCF-7 or MDA-MB-231) in each group were prepared into 100 μL cell suspension (1 × 10^4^ cells/mL) and seeded into 96-well plates. After incubation at 37°C and 5% CO_2_ condition for 0, 24, 48, and 72 h, 10 μL CCK-8 solution (Dojindo, Kumamoto, Japan) was added to each well and mixed. Next, the cells were incubated in a temperature box for 2 h, and the absorbance value (450 nm) was measured using a microplate reader.

### Cell motility experiments

The migration and invasion abilities of transfected cells were evaluated using a 24-well Transwell chamber (Corning, NY, USA) with a pore size of 8 μm. Matrigel matrix (BD Science, Sparks, MD, USA) was used to pre-coat after premelting at 4°C for the invasion assay but not the migration assay. A total of 100 μLof treated MCF-7 and MDA-MB-231 cells were seeded in 24-well plates at a density of 1 × 10^5^ cells per well, and 600 μL of complete medium were seeded under the wells. Cells that did not migrate or invade the surface of the lower chamber after 24 h were wiped clean with cotton swabs, while cells that migrated and invaded the surface of the lower chamber were stained with crystal violet for 10 min. Finally, the number of stained cells was observed under a light microscope and counted.

### Dual-luciferase reporter assay

The circular RNA interactome online database was used to identify miRNAs targeting has_circ_0000069 [21]. The luciferase reporter vectors has_circ_0000069 wide type (wt-circRNA) containing miR-432 binding sites and has_circ_0000069 mutant type (mut-circRNA) were constructed for dual-luciferase reporter assay. The luciferase reporters were cloned into pmirGLO Dual-Luciferase vectors. Lipofectamine 2000 was used to transfect the luciferase vector and miR-432 mimics/mimic NC into MCF-7 and MDA-MB-231 cells. After 24 h of culture, the luciferase activities were detected with GloMax 20/20 luminescence detector.

### Statistical analysis

SPSS software (version 20.0) and GraphPad software (version 7.0) were used for data analysis. Measurement data were expressed as mean ± SD. Comparison between two groups was performed by student’s *t*-test. Comparison among three or more groups were performed using one-way ANOVA. The correlation between circRNA expression and the prognosis of breast cancer patients was analyzed by Kaplan-Meier survival analysis. *p* < 0.05 was considered statistically significant.

## Results

### Has_circ_0000069 expression in breast cancer was related to several clinical parameters

**FIGURE 1:**
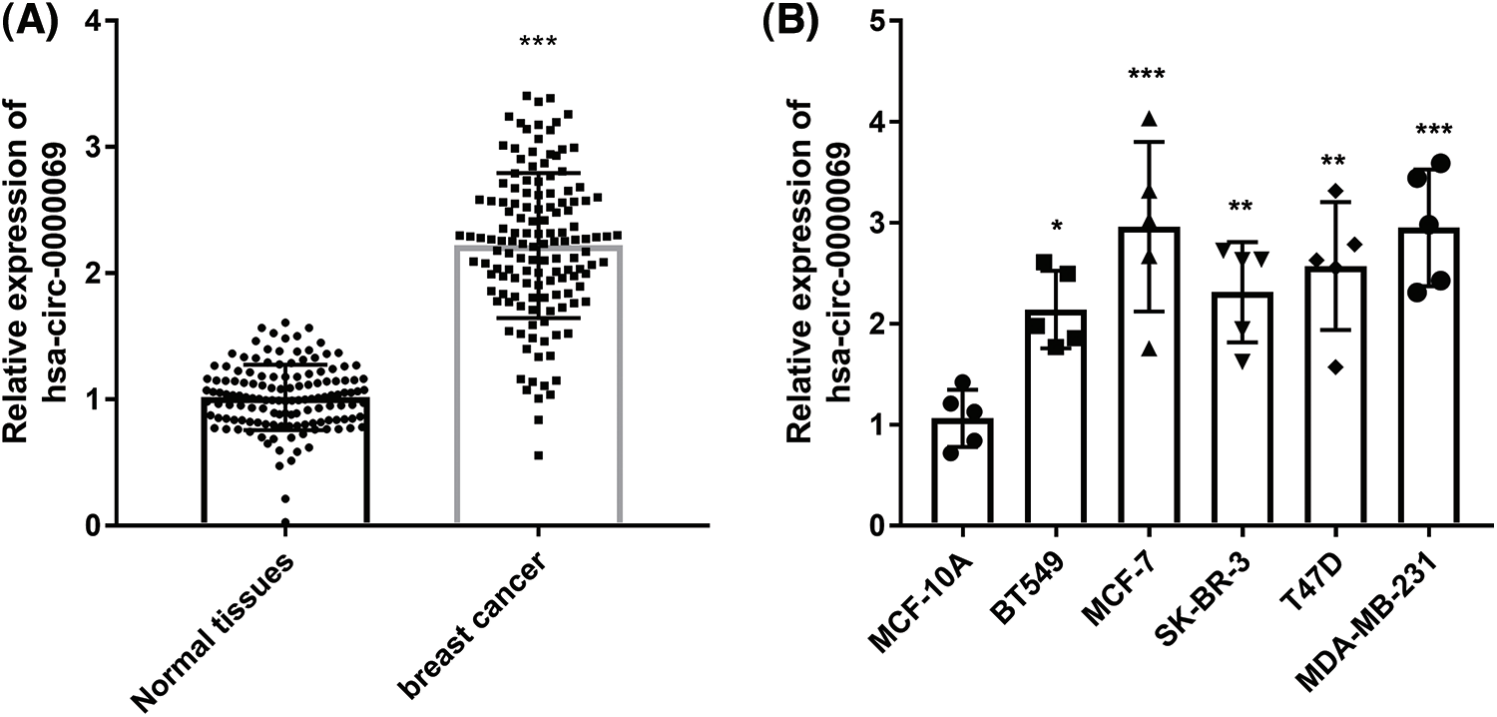
Has_circ_0000069 expression increased in breast cancer. (A) Relative expression levels of has_circ_0000069 were measured in 137 pairs of tissue specimens. (B) Has_circ_0000069 expression levels were higher in breast cancer cells than normal breast epithelial cell lines MCF-10A (n = 5). *p < 0.05, **p < 0.01, ***p < 0.001.

The expression of has_circ_0000069 in breast cancer was assessed in tissue specimens. RT-qPCR revealed that has_circ_0000069 expression was raised in breast cancer tissues compared with normal tissues (*p* < 0.001, Fig. 1A). Moreover, has_circ_0000069 levels were higher in different cancer cells BT549 (ER/PR/HER2-negative), MCF-7 (ER+), SK-BR-3 (HER+), T47D (ER+), MDA-MB-231 (ER/PR/HER2-negative), than normal breast cell MCF-10A (*p* < 0.05, Fig. 1B). Among these breast cancer cells, MCF-7 and MDA-MB-231 cells showed higher has_circ_0000069 expression levels, which were used in subsequent experiments.

In addition, the average expression value of has_circ_0000069 (2.219) in all tumor tissues was used as the cut-off value to group patients into low circ_0000069 expression group and high has_circ_0000069 expression group. Subsequently, the correlation between has_circ_0000069 expression and clinical parameters of patients was analyzed. The results in Table 1 show that a high expression of circ_000069 was related to patients’ clinical stage (*p* = 0.007) and lymph node metastasis (*p* = 0.015).

**TABLE 1:** Correlation between circ-0000069 expression and clinical characteristics of breast cancer patients

Parameters	Total (n)	Circ_0000069 expression		*p* value
		Low (n = 65)	High (n = 72)	
Age (years)				0.942
≤55	70	33	37	
>55	67	32	35	
ER status				0.234
Negative	47	19	28	
Positive	90	46	44	
PR status				0.359
Negative	41	17	24	
Positive	96	48	48	
HER2 status				0.124
Negative	99	51	48	
Positive	38	14	24	
Ki67				0.059
<14%	56	32	24	
≥14%	81	33	48	
Clinical stage				0.007
I–II	92	51	41	
III	45	14	31	
Lymph node metastasis				0.015
Negative	89	49	40	
Positive	48	16	32	

Note: ER: estrogen receptor, PR: progesterone receptor, HER2: human epidermal growth factor receptor 2.

### The increased has_circ_0000069 expression predicted poor overall survival of patient

**FIGURE 2:**
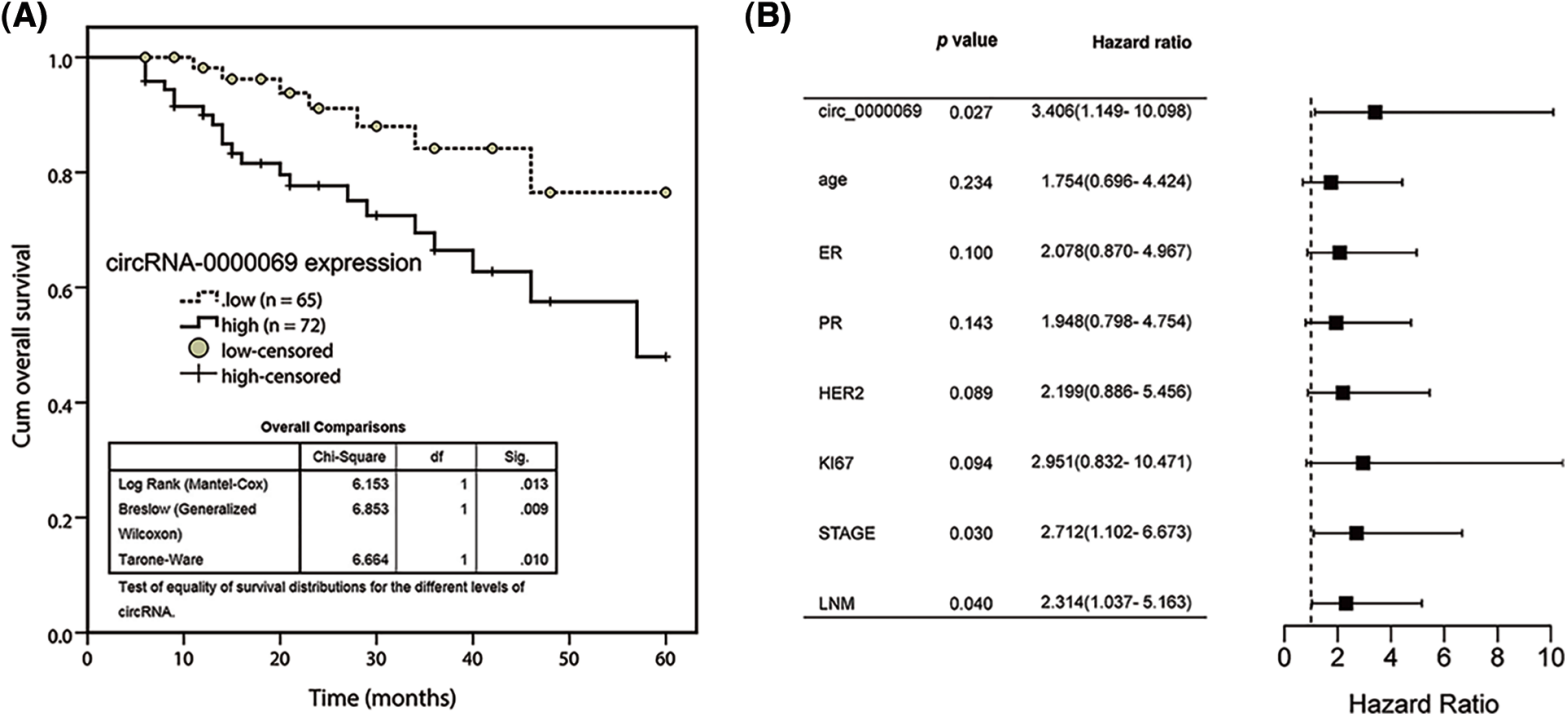
Kaplan-Meier survival curve was conducted to evaluate the prognostic value of has_circ_0000069 in breast cancer. (A) Increased has_circ_0000069 was linked to the shorter overall survival rate of breast cancer patients. Log-rank test *p* = 0.013. (B) Forest plot for multivariate Cox regression analysis.

The clinical value of has_circ_0000069 expression was evaluated and the survival curve showed that high has_circ_0000069 contributed to the shorter overall survival time of breast cancer patients (*p* = 0.013, Fig. 2A). Multiple Cox regression analysis was carried out to explore prognostic risk factors associated with patient’s prognosis. The analysis results revealed that has_circ_0000069 expression (*p* = 0.027), clinical stage (*p* = 0.030), and lymph node metastasis (*p* = 0.040) were prognostic risk factors (Fig. 2B).

### Transfection efficiency was detected

**FIGURE 3:**
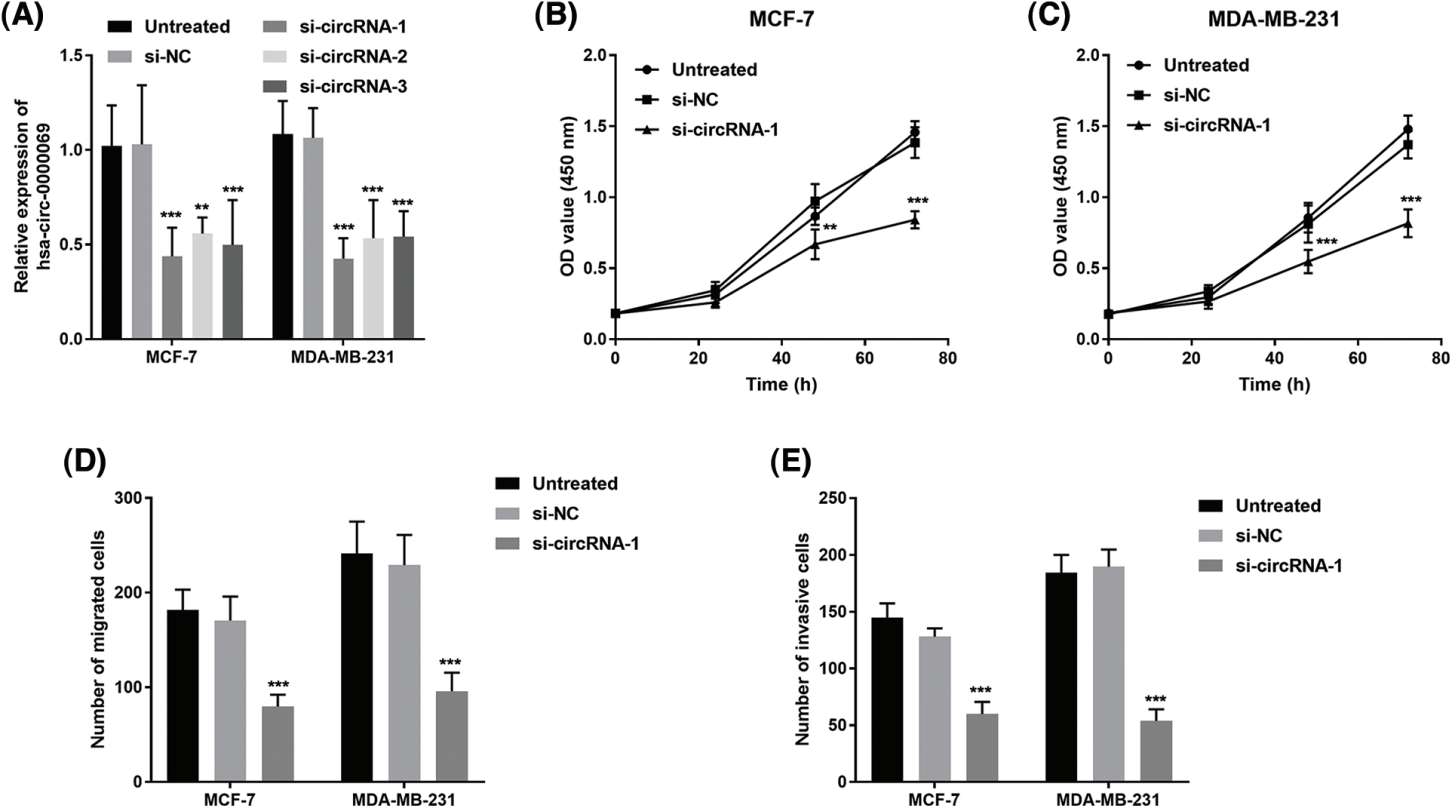
Silencing has_circ_0000069 decreased the proliferation, migration, and invasion potential in MCF-7 and MDA-MB-231 cells. (A) si-circRNAs decreased the has_circ_0000069 levels (n = 5). (B) and (C) CCK-8 assay was used to detect the influence of has_circ_0000069 on cell proliferation (n = 3). (D) The cell migration abilities were weakened by si-has_circ_0000069 (n = 5). (E) Knockdown of has_circ_0000069 decreased the cell invasion capacities (n = 5). **p < 0.01, ***p < 0.001.

The has_circ_0000069 expression was knocked down (si-circRNA-1, si-circRNA-2, and si-circRNA-3) for the investigation of the biological role of has_circ_0000069 in MCF-7 and MDA-MB-231 cells, which are high has_circ_0000069 expressed cell lines. The transfection verification results showed si-circRNA-1 significantly decreased the has_circ_0000069 levels in cancer cells (*p* < 0.001, Fig. 3A), and these were used in the following experiments.

### Has_circ_0000069 facilitates cellular abilities in breast cancer cells

CCK-8 assay revealed that has_circ_0000069 knockdown repressed the growth of treated cells compared with untreated cells (*p* < 0.05, Figs. 3B and 3C). In addition, the role of has_circ_0000069 on the migration capacities and invasion potential of breast cancer cells was investigated using Transwell assays. As shown in Figs. 3D and 3E, silencing has_circ_0000069 decreased the capacities of breast cancer cell migration and invasion (*p* < 0.01).

### Has_circ_0000069 functions as a ceRNA of miR-432 in breast cancer cells

**FIGURE 4:**
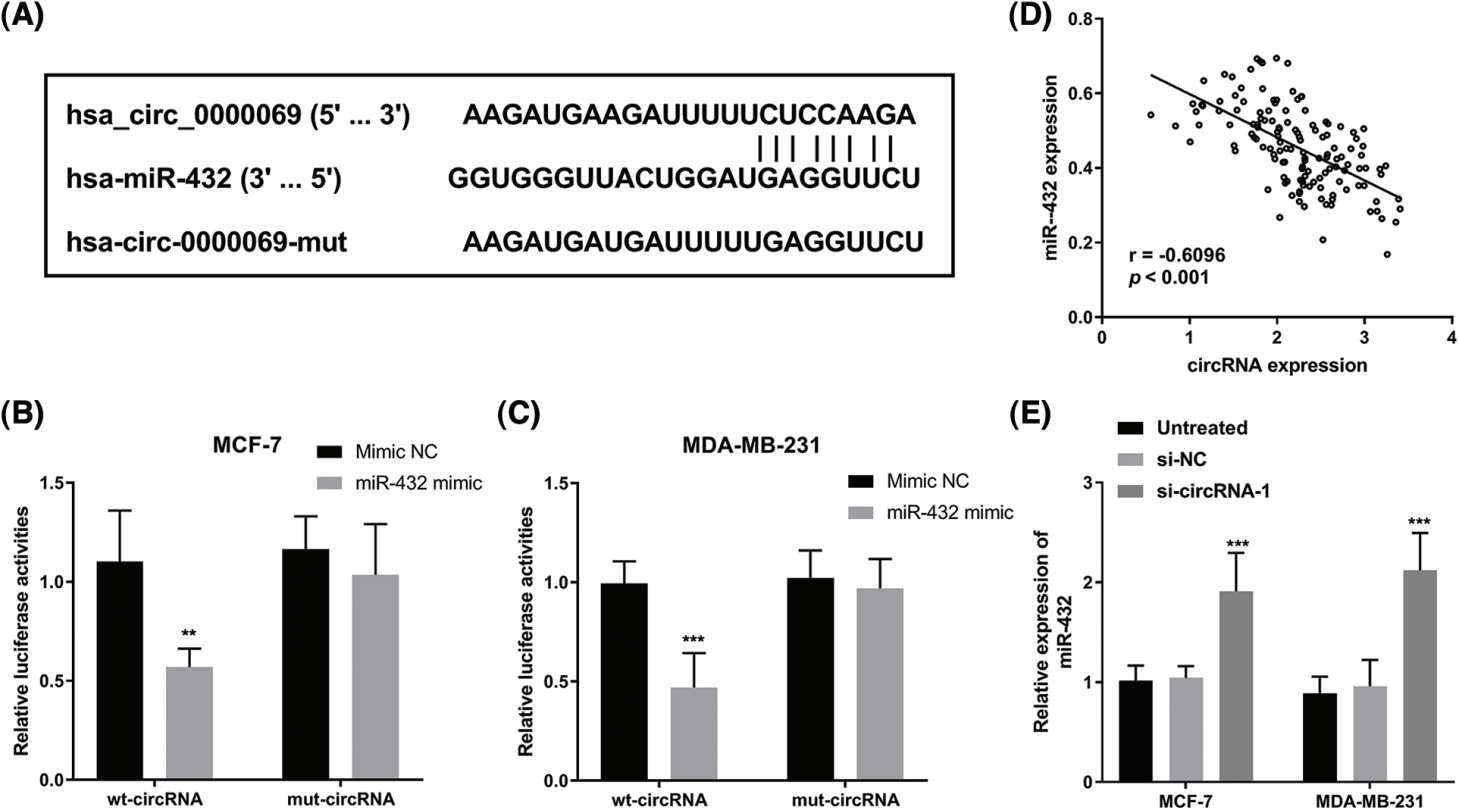
Has_circ_0000069 interacted with miR-432. (A) The binding sites between has_circ_0000069 and miR-432. (B) and (C) Luciferase reporter assay of MCF-7 and MDA-MB-231 cells transfected with wt-circRNA or mut-circRNA together with miR-432 mimic or mimic NC (n = 5). (D) Pearson correlation between has_circ_0000069 and miR-432 expression in tumor tissues (n = 137). r = −0.6096, *p* < 0.001. (E) The expression of miR-432 increased in has_circ_0000069- silence cells (n = 5). **p < 0.01, ***p < 0.001.

The online website circular RNA interactome (https://circinteractome.nia.nih.gov/index.html) was used to predict putative miRNAs interacted with has_circ_0000069. Based on the search results, we selected miRNAs with context+ score percentile more than 98, which include miR-1253, miR-548c-3p, miR-873, miR-940, and miR-432 (Suppl. Table 1). Among these miRNAs, miR-432 had the highest context+ score (−0.416), which was further verified in this study. The binding sites between has_circ_0000069 and miR-432 are shown in Fig. 4A. We verified that a higher expression of miR-432 led to a decrease in luciferase activity of the wt-circRNA vector in MCF-7 (Fig. 4B) and MDA-MB-231 cells (Fig. 4C). The Pearson correlation analysis showed the miR-432 and has_circ_0000069 have a negative correlation (r = −0.6096, *p* < 0.001, Fig. 4D). Then, miR-432 levels were detected in si-circRNA-1 transfected breast cancer cells. miR-432 levels were increased in MCF-7 and MDA-MB-231 cells with downregulated-has_circ_0000069 expression (*p* < 0.001, Fig. 4E).

**FIGURE 5:**
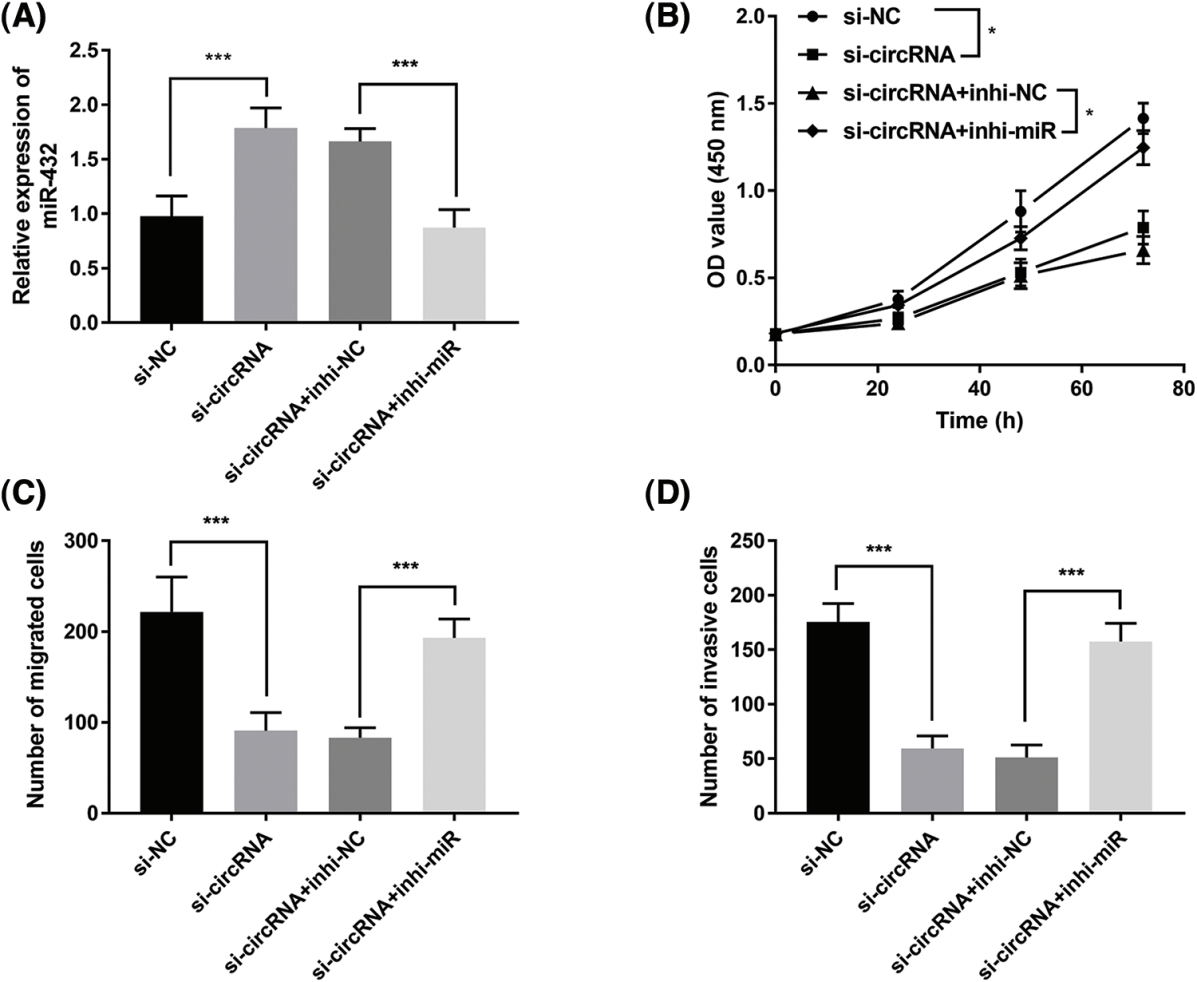
Downregulation of miR-432 promotes breast cancer cellular activities and reverses the inhibition effects of si-circRNA in MDA-MB-231 cells. (A) The efficiency of miR-432 downregulation in MDA-MB-231 cells (n = 5). (B) CCK-8 assay measured the effects of miR-432 on cell proliferation rate (n = 3). (C) The migration capacities of MDA-MB-231 cells were evaluated using Transwell assay (n = 5). (D) The invasion abilities were verified by Transwell invasion assay (n = 5).

Moreover, we investigated whether miR-432 was a functional target of has_circ_0000069 in MDA-MB-231 cells. The RT-qPCR assay showed that miR-432 expression was upregulated in si-circRNA-transfected MDA-MB-231 cells, while miR-432 inhibitor decreased miR-432 expression (*p* < 0.001, Fig. 5A). The CCK-8 assay showed that silencing miR-432 reversed the decreased cell proliferation ability by downregulation of has_circ_0000069 (*p* < 0.05, Fig. 5B). Moreover, Transwell assays pointed that a low expression of miR-432 motivated the migration (Fig. 5C) and invasion abilities (Fig. 5D) of MDA-MB-231 cells (*p* < 0.001). The rescue experiments showed downregulation of miR-432 could attenuate the function of has_circ_0000069 siRNA in breast cancer cells (Figs. 5B–5D).

## Discussion

The present study verified the high has_circ_0000069 expression in 137 pairs of breast cancer tissues and normal tissues. The increased has_circ_0000069 expression was linked to the shorter overall survival of patients. Downregulation of has_circ_0000069 receded breast cancer cellular behaviors by targeting miR-432. These data reveal that targeting has_circ_0000069 may represent a prognostic predictor and therapeutic target for treating patients with breast cancer.

Emerging evidence has shown that circRNAs play impotant roles in tumor progression [22]. Many circRNAs function as oncogene or tumor suppressors in breast cancer [23,24]. For instance, has_circ_102229 was upregulated in triple-negative breast cancer tissues and associated with poor prognosis, as well as could facilitate tumor progression by regulating miR-152-3p/PFTK1 pathway [25]. A recent study identified circRNA-miRNA-mRNA network and prognostic prediction in breast cancer, in which has_circ_0000069 was an upregulated circRNA [20]. Consistent with the expression pattern, has_circ_0000069 expression was upregulated in breast cancer tissues in this study. These findings suggest that has_circ_0000069 may play an oncogenic role in breast cancer. Moreover, has_circ_0000069 levels were also higher in breast cancer cells than in normal breast cells. However, we did not find a significant difference among different cell lines, including BT549 (ER/PR/HER2-negative), MCF-7 (ER+), SK-BR-3 (HER+), T47D (ER+), MDA-MB-231 (ER/PR/HER2-negative), which suggest that has_circ_0000069 may have no significant difference among different types of breast cancer.

Has_circ_001783 [15], has_circ_102229 [25], and has_circ_0000515 [26] expression in tissues were associated with the prognosis of breast cancer patients. Therefore, to clarify the clinical significance of has_circ_0000069 in breast cancer, the association analysis between has_circ_0000069 expression and patients’ clinical characteristics was analyzed. The results indicated that has_circ_0000069 expression was related to positive lymph node metastasis and clinical stage. Patients with high has_circ_0000069 expression had shorter overall survival. Similar to our findings, upregulation of has_circ_0000069 was closely related to poor prognosis of cervical cancer [27] and pancreatic cancer patients [18]. Moreover, has_circ_0000069 expression was an independent prognostic risk factor in breast cancer. These data indicate that has_circ_0000069 expression in breast cancer is negatively correlated with the prognosis, and it may be acted as a potential predictor to determine the prognosis of breast cancer patients.

Many circRNAs come into working with a tumor-promoting role in breast cancer and are involved in tumorigenesis by modulating miRNAs [28–30]. For instance, circACAP2 facilitates breast cancer growth and motility by regulating miR-29a/b and modulating COL5A1 [31]. High has_circ_0000069 expression was reported in colorectal cancer and accelerated cellular functions in colorectal cancer progression [19]. In this study, it is observed that has_circ_0000069 is highly expressed in breast cancer, however, its function remains unclear. Further loss-functional research revealed that silencing has_circ_0000069 could repress breast cancer cell proliferation and motility. Thus, has_circ_0000069 may play a tumor-promoting role in breast cancer progression. The previous circRNA-miRNA-mRNA regulatory network showed has_circ_0000069-miR-125b-5p-EIF4EBP1 in the PPI network in breast cancer [20]. Whereas, we predicted that several miRNAs have binding sites with has_circ_0000069 and verified that has_circ_0000069 could target miR-432. Downregulation of miR-432 could attenuate the function of has_circ_0000069 siRNA in breast cancer cells. miR-432 was downregulated in breast cancer tissues and functioned as a tumor inhibitor in breast cancer by targeting E2F3 or SLBP [32–34]. These data showed that has_circ_0000069 might facilitate breast cancer progression by regulating miR-432 then modulating E2F3 or SLBP expression.

In all, we verified that has_circ_0000069 expression increases in breast cancer tissues and is associated with patients’ overall survival. Knockdown of has_circ_0000069 could reduce the cellular capacities of progression, migration, and invasion of breast cancer cells by targeting miR-432. Therefore, targeting has_circ_0000069 may be a potential prognostic predictor and therapeutic target for treating breast cancer patients.

## Data Availability

Corresponding author may provide data and materials.

## Appendix

**SUPPLEMENTARY TABLE 1:** Has_circ_0000069-related miRNAs predictions

CircRNA mirbase ID	CircRNA start	CircRNA end	Context+ score percentile	Context+ score
miR-1253	440	447	98	−0.225
miR-548c-3p	68	74	98	0.120
miR-873	137	144	99	−0.353
miR-940	392	399	99	−0.324
miR-432	75	82	99	−0.416
